# *In Vitro* Biocompatibility and Endothelialization of Novel Magnesium-Rare Earth Alloys for Improved Stent Applications

**DOI:** 10.1371/journal.pone.0098674

**Published:** 2014-06-12

**Authors:** Nan Zhao, Nevija Watson, Zhigang Xu, Yongjun Chen, Jenora Waterman, Jagannathan Sankar, Donghui Zhu

**Affiliations:** 1 Department of Chemical, Biological and Bio-Engineering, North Carolina Agricultural and Technical State University, Greensboro, North Carolina, United States of America; 2 NSF Engineering Research Center-Revolutionizing Metallic Biomaterials, North Carolina Agricultural and Technical State University, Greensboro, North Carolina, United States of America; 3 Center for Advanced Materials and Smart Structures (CAMSS), Mechanical Engineering, North Carolina Agricultural and Technical State University, Greensboro, North Carolina, United States of America; 4 Animal Science, North Carolina Agricultural and Technical State University, Greensboro, North Carolina, United States of America; University of Akron, United States of America

## Abstract

Magnesium (Mg) based alloys are the most advanced cardiovascular stent materials. This new generation of stent scaffold is currently under clinical evaluation with encouraging outcomes. All these Mg alloys contain a certain amount of rare earth (RE) elements though the exact composition is not yet disclosed. RE alloying can usually enhance the mechanical strength of different metal alloys but their toxicity might be an issue for medical applications. It is still unclear how RE elements will affect the magnesium (Mg) alloys intended for stent materials as a whole. In this study, we evaluated MgZnCaY-1RE, MgZnCaY-2RE, MgYZr-1RE, and MgZnYZr-1RE alloys for cardiovascular stents applications regarding their mechanical strength, corrosion resistance, hemolysis, platelet adhesion/activation, and endothelial biocompatibility. The mechanical properties of all alloys were significantly improved. Potentiodynamic polarization showed that the corrosion resistance of four alloys was at least 3–10 times higher than that of pure Mg control. Hemolysis test revealed that all the materials were non-hemolytic while little to moderate platelet adhesion was found on all materials surface. No significant cytotoxicity was observed in human aorta endothelial cells cultured with magnesium alloy extract solution for up to seven days. Direct endothelialization test showed that all the alloys possess significantly better capability to sustain endothelial cell attachment and growth. The results demonstrated the promising potential of these alloys for stent material applications in the future.

## Introduction

Magnesium (Mg) biodegradable material is a promising new generation of candidate for vascular stents. The key advantage of Mg-based material over others for stent material is its potential to significantly reduce or even eliminate the late restenosis which occurs very frequently in permanent stent materials [1]–[3]. Pure Mg has favorable biocompatibility but the mechanical strength and corrosion resistance cannot meet the requirement of vascular stents. Therefore, numerous Mg-based alloys have been explored to improve the mechanical and corrosion properties while maintain the good biocompatibility.

Various new Mg alloys such as MgZn [4], MgZnCa [5], [6], MgAlZn [7], Mg-RE [8], [9] and others were developed in the past. For example, the ultimate tensile strength (UTS) of extruded Mg-Al-Ca-Mn alloy was about 420 MPa, whereas the elongation was sharply reduced to 5% [10]. Mao et al. showed corrosion behavior of as-cast Mg-Nd-Zn-Zr was significantly improved [11]. Another study reported that binary alloy Mg-0.5Sr significantly decreased the corrosion rate in simulated body fluid (SBF), which is about 1.63 mg/(d*cm^2^), and the in vivo study using canine model showed that the stent with the strut of 0.27 mm could last 35–38 weeks [12], still not long enough for certain clinical applications. Zhou et al. demonstrated that Mg-Li-Al-RE alloys with increased mechanical strength had no obvious effects on cell viability [13]. Despite these successes, the gap between material development and clinical demand for ideal stents still exists.

Among all of those alloying elements, RE elements were demonstrated great efficiency in increasing the yield strength (YS), elongation and corrosion resistance of alloys by refining the grain structure and reducing the grain boundary [14]–[19]. The mechanical strength, corrosion behavior and biocompatibility of other alloys including Mg-Y, Mg-Nd-Y, Mg-Zn-Y, Mg-Zn-Y-Nd, Mg-Nd-Zr, and Mg-Nd-Zn-Zr were also investigated before [11], [20]–[24]. Meshinchi et al. showed the addition of RE elements to AZ91 alloy can increase its ultimate tensile stress from 137 to 166 MPa [25]. The UTS of Mg-Li-(Al)-(RE) alloys were also increased up to 234 MPa and the elongation was up to 46.1% [1]. Mg-3Al-RE fabricated by Tian et al. demonstrated an improved UTS of 201 MPa but a relative low elongation of 8.2% [26]. Although some previous studies have shown that Mg-RE based vascular stents have no significant cytotoxic effect [27], [28], there is still a great deal of concerns on the potential toxicity of RE elements. In the efforts of continuing search for ideal vascular stent biomaterials, four Mg-RE alloys were designed and fabricated in our lab. Their mechanical properties, crystal structure, corrosion resistance, hemocompatibility, and endothelialization were studied to evaluate their feasibility and potential as new biodegradable stent materials.

## Materials and Methods

### Material preparation

Mg based alloys of MgYZr-1RE, MgZnYZr-1RE (RE includes Gd and Dy, denoted as R1 and R2, respectively, thereafter), as well as MgZnCaY-*x*RE (*x* = 1 and 2, RE includes ND and Gd, denoted as R3 and R4, respectively, thereafter) were prepared by melting 99.97% Mg ingot (Alfa Aesar, US), 99.9% Zn, Ca granules (Sigma-Aldrich, US) and Mg-30RE master alloys in an electrical resistance furnace (Mbraun, US) under the protection of argon gas. Mg-30RE is a gift from Institute of Metal Research, Chinese Academy of Science. The melt was stirred using a power stirring tool at 300 rpm for 20 min to homogenize the alloy. Mg-30Zr master alloy was added into the melt at last after all the other alloying elements and master alloy were thoroughly melted. Afterwards, the melt was kept still for 20 min and the temperature was raised to 730°C before casting [29]. The melt was cast in a permanent low-carbon steel book mold which was heated to about 300°C.

All the materials were then cut (Techcut 5, Allied High Tech Products, US) into 10×10×1 mm square and polished (EcoMet 250 Grinder, Buehler, US) with SiC paper up to 1200 grit. All samples were supersonically cleaned (M2510 Ultrasonic cleaner, Branson, US) in acetone and ethanol. For hemocompatibility and endothelialization tests, each side of the samples was sterilized by UV light (1380 Biological Safety Cabinet, Thermo, US) exposure for 30 min. At least 3 replicates were used in each experiment (n≥3).

### Microstructure characterization

For microstructural studies, specimens were polished and etched in a 5 vol% nital solution. Microstructure was examined by an optical microscope (Axio Imager M2M, Zeiss, US).

### Mechanical property characterization

The samples for tensile test were cut from the same area of the ingot by Electric Discharge Machining (Instron 5566, Instron Corporation, US). The width of the samples was 3 mm and the gauge length was about 12.5 mm which is proportional to the dimensions described in the ASTM standard B557M-07. The tests were conducted under rate control at 1 mm/min in ambient environment.

### Electrochemical corrosion

Potentiodynamic polarization curves were measured by Gamry instruments (Gamry Ref 600, Gamry Instruments, US) using a three-electrode cell as described previously [30]. The reference electrode, counter electrode and working electrode were saturated calomel, platinum and testing materials. Tests were carried out in Hank's balanced solution (HBS, Invitrogen, US) after samples were immersed for a while. All the DC polarization data was fitted and analyzed by Echem Analyst 6.0 (Gamry instrument, US). The corrosion rate of all the samples were calculated according to ASTM-G102-89 [31].

### Hemolysis

Fresh human whole blood with sodium citrate was purchased from Cedarlane Labs (Cedarlane, US). The test was performed according to the method described previously [4]. In brief, 0.2 ml diluted human whole blood (4∶5) was added to the 10 ml centrifuge tubes after all the samples were pre-dipped into 10 ml Dulbecco's Phosphate Buffered Saline (DPBS, Invitrogen, US) for 1 hour. The positive and negative groups were diluted blood with 10 ml deionized water and diluted blood with 10 ml DPBS, respectively. Then all the samples were incubated at 37°C for 1 hour. After centrifuge (Biofuge Stratos, Thermo, US) at 800 g for 5 min, the supernatants were collected and the absorbance (A) was measured by UV-Vis Spectrometer (Thermo, US) at 545 nm. The hemolysis rate (HR) was calculated by the following equation:
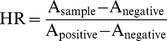



### Platelet adhesion

Platelet rich plasma (PRP) with platelets of 10^8^/ml (All Cells, US) was used for platelet adhesion test. All the samples were pre-soaked in DPBS for 1 hour. Then 100 µl PRP was overlaid on the surface of each sample and incubated (Heracell 150 I, Thermo, US) at 37°C for 1 hour. Samples were gently rinsed by DPBS for 3 times to remove the non-adherent platelets. After that, 4% paraformaldehyde (Boston Bioproducts, US) was used to fix the adherent platelets followed by ethanol gradient (50%, 60%, 70%, 80%, 90%, and 100%) dehydration for 10 minutes. All the samples were coated by gold nanoparticle for 2 min and observed by SEM (SU8000, Hitachi, US). The number of adherent platelets was counted by Image-Pro Plus 6.0 (Media Cybernetics, US) on at least four different SEM images for each sample.

### Indirect cytocompatibility assessments

Human aorta endothelial cells (HAEC) were purchased from Sciencell Research Laboratories (California, US). HAECs were expanded in endothelial culture medium (ECM, Sciencell, US) with 10% fetal bovine serum (Sciencell, US), 100 U/ml penicillin (Sciencell, US) and 100 µg/ml streptomycin (Sciencell, US) on the fibronectin coated 75-flasks (BD Biosciences, US) at 37°C in humidified incubator with 5% CO_2_ and the passages 4–5 were used. Indirect MTT (Invitrogen, US) test was used to measure cell toxicity. Mg extracts were prepared according to ISO 10993-12 [32]. Samples were soaked with serum free ECM as the extraction medium with a ratio of 1.25 ml/cm^2^ in a humidified atmosphere with 5% CO_2_ at 37°C for 3 days. The supernatant was removed, centrifuged, filtered and refrigerated at 4°C. HAECs were seeded in the 96-well cell culture plate (BD Bioscience, US) for 24 hours to allow cell attachment. ECM then was replaced by 100 µl 10%, 25%, 50%, and 75% extract solutions. The positive control and negative control were serum free ECM and serum free ECM with 10% DMSO (Invitrogen, US), respectively. After incubating in the humidified incubator for 2, 4, and 7 days, MTT test was performed according to the manufacturer's protocol. The absorbance (A) was measured at 570 nm by a Microplate reader (SpectraMax, Molecular Devices, US). Morphology of cells was characterized by Digital Inverted Microscope (EVOS, Advanced Microscopy, US). Cell viability was calculated by the following equation: 
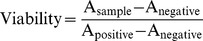



### In vitro endothelialization measurement

Mg alloy samples were soaked into serum free ECM for 3 days in 24-well culture plate (BD Bioscience, US) before using. Cell suspension (100 µl) with density of 100,000 cell/100 µl was overlaid on the surface of samples. Fibronectin coated tissue culture plate was used as control group. Cells were allowed to settle down for a while in an incubator with 5% CO_2_ at 37°C. DPBS was used to gently wash the sample surface for 3 times to remove the unattached cells. And then 2 ml serum free ECM was added to each well. After 3 hours and 24 hours, Mg samples were transferred to another new plate and cells on the sample surface were characterized by LIVE/DEAD Viability Kit (Invitrogen, US) according to the manufacturer's protocol. Images were taken by Digital Inverted Microscope (EVOS, US). The culture media was transferred to centrifuge tube (Fisher Scientific, US) and centrifuged at 8,000 g for 5 min. Then the supernatant was used for pH measurement (2100 Series, Oaklon, US).

### Statistical analysis

All data was expressed as Mean±SD. Statistical analysis was performed in Prism 5 software (GraphPad, US). One-way ANOVA was used to test significant difference in hemolysis and cell viability. Unpaired two-tailed t test was used to compare difference between two groups. It is considered significantly different statistically if the P<0.05.

## Results

### Alloy microstructure

**Figure 1:**
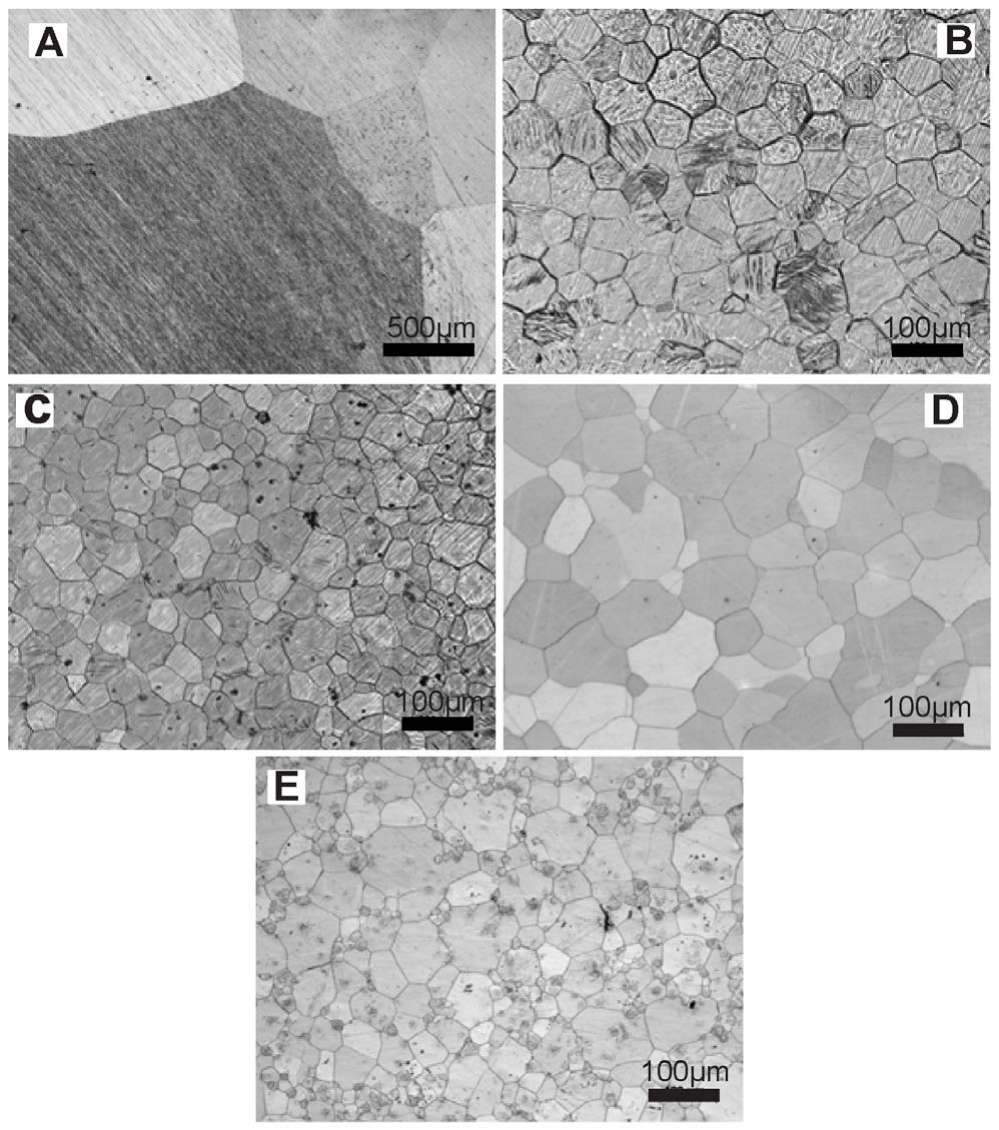
Representative optical microscope images of alloy microstructures. (A) Pure Mg, and (B–E) R1–R4.

The typical optical microstructure of pure Mg, R1, R2, R3, and R4 were characterized in Fig. 1. Pure Mg had relative big non-uniform grain structure with the size ranging from five hundred to thousands microns. The uniform equiaxed grains displayed in R1 had the average grain size about 47.6 µm and a few transparent particles appeared in some of the α-phase. The grain boundary in R1 was the most obvious compared with other three materials. The average grain size of R2 was about 35.7 µm and more dark spots were observed in R2. In comparison, the average grain size in R3 was about 66.7 µm, the biggest among all the four alloys. Average grain size in R4 was about 40.0 µm including substantial amount of polygon second phases.

### Alloy mechanical properties

**Figure 2:**
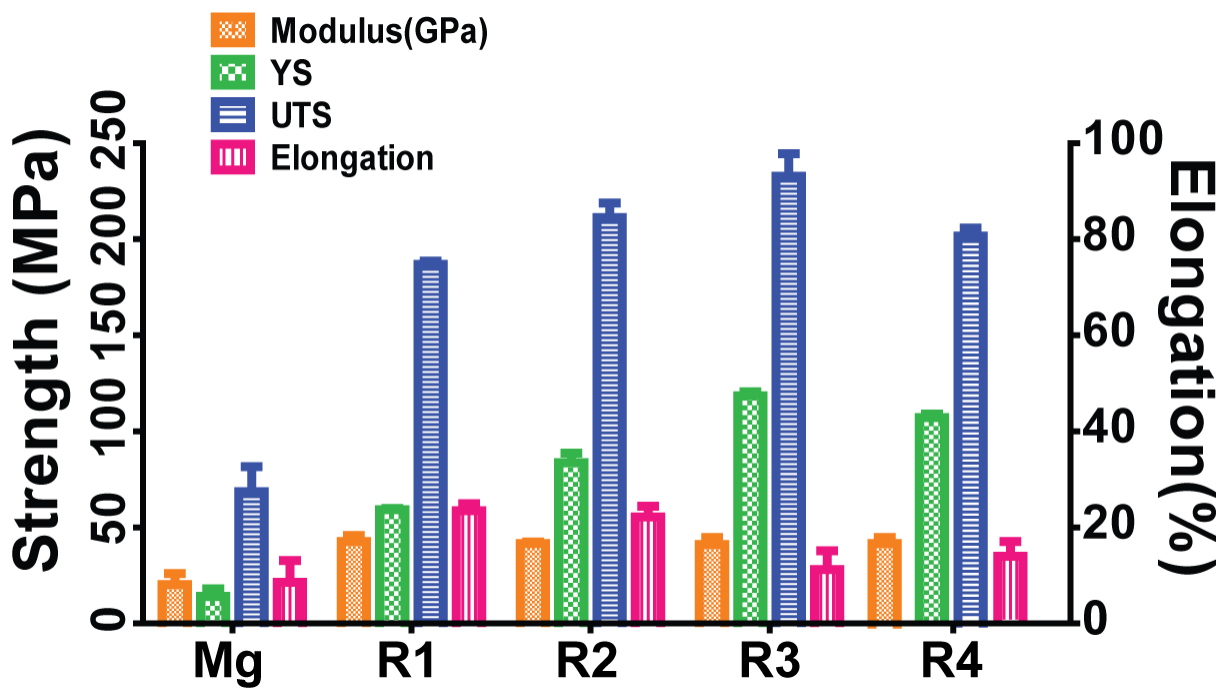
Mechanical strength of pure Mg and Mg-RE alloys at room temperature.

The mechanical properties of the tested materials were shown in Fig. 2. All the mechanical properties of the alloys including the modulus, YS, UTS, and elongation were significantly improved compared with that of pure Mg. The YS of the alloys varied between 59.3 to 118.5 MPa, and the UTS of RE alloys were between 187.0 to 232.8 MPa. R3 had the highest YS and UTS. R1 and R2 also exhibited most significantly enhanced elongation. The YS and UTS of R3 were higher than that of R4 while the elongation and modulus were lower than that of R4.

### Alloy corrosion properties

**Figure 3:**
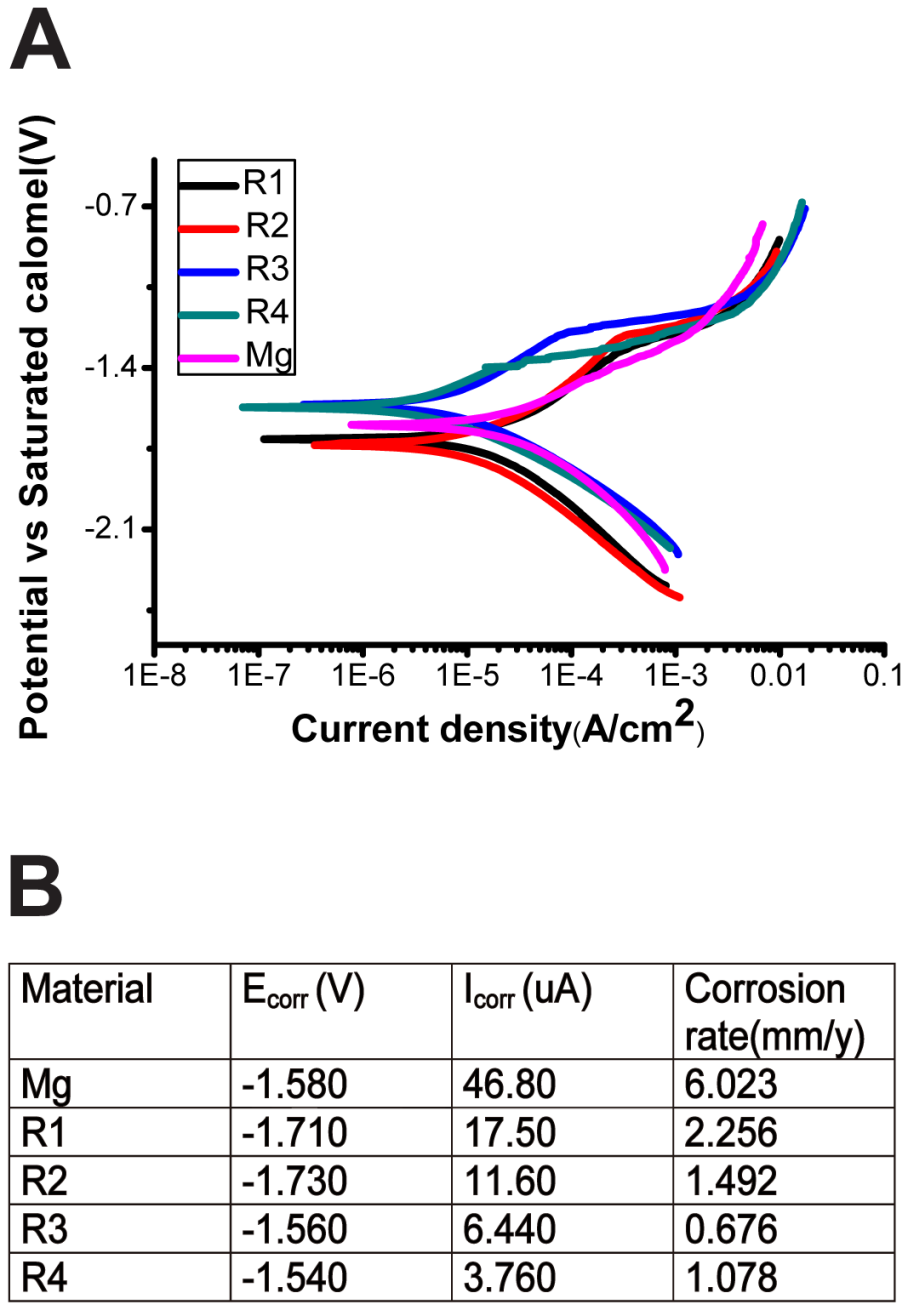
Corrosion rate of Pure Mg and Mg-RE alloys. (**A**) Potentiodynamic polarization curves in HBS, and (**B**) Electrochemical corrosion data of Pure Mg and Mg alloys.

Potentiodynamic polarization curves of four RE alloys and pure Mg in HBS were shown in Fig. 3A. An obvious shift of E_corr_ in cathodic direction and reduction in cathodic current density were observed in R3 and R4. The current density in all alloys decreased significantly compared with pure Mg. The E_corr_, I_corr_ and corrosion rate of all Mg materials were summarized in Fig. 3B. The corrosion rates of three RE alloys were 3 to 10 times lower than that of pure Mg.

**Figure 4:**
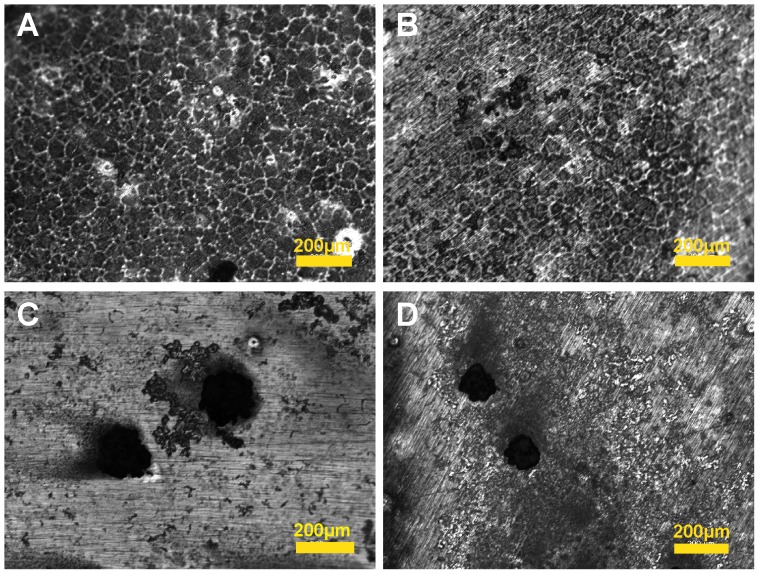
Topographical changes of Mg-RE alloys after DC polarization. (**A–D**) **R1–R4.**

The representative morphology of alloys after DC polarization was shown in Fig. 4. In all RE alloys, homogeneous corrosion was dominant. Intergranular corrosion and a few pitting corrosion of 10 to 50 µm in diameter were present in R1 and R2. In R3 and R4, large pitting corrosion was observed, consistent with the results from the DC polarization curves.

### Hemolysis

**Figure 5:**
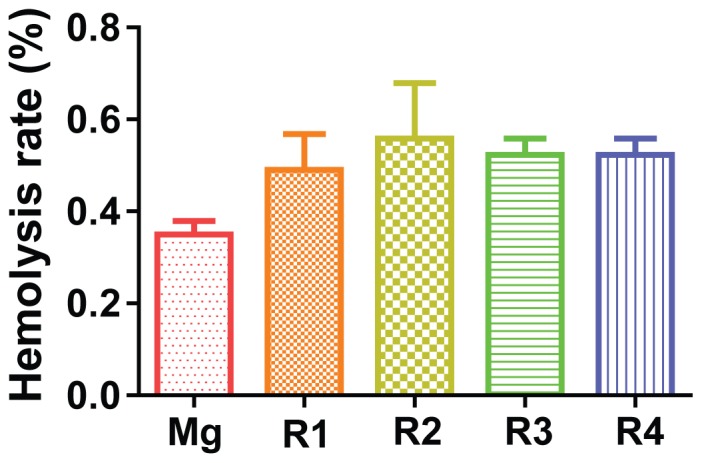
Hemolysis rate of diluted human whole blood after incubated with Mg materials for 1

The hemolysis rate (HR) of the materials was shown in Fig. 5. All the hemolysis rates of the materials were smaller than 0.6%, much lower than the 5% threshold, therefore, all samples were considered non-hemolytic according to the ISO 10993-4:2002 standard [33], [34]. There was no significant difference among all the Mg materials tested.

### Platelet adhesion

**Figure 6:**
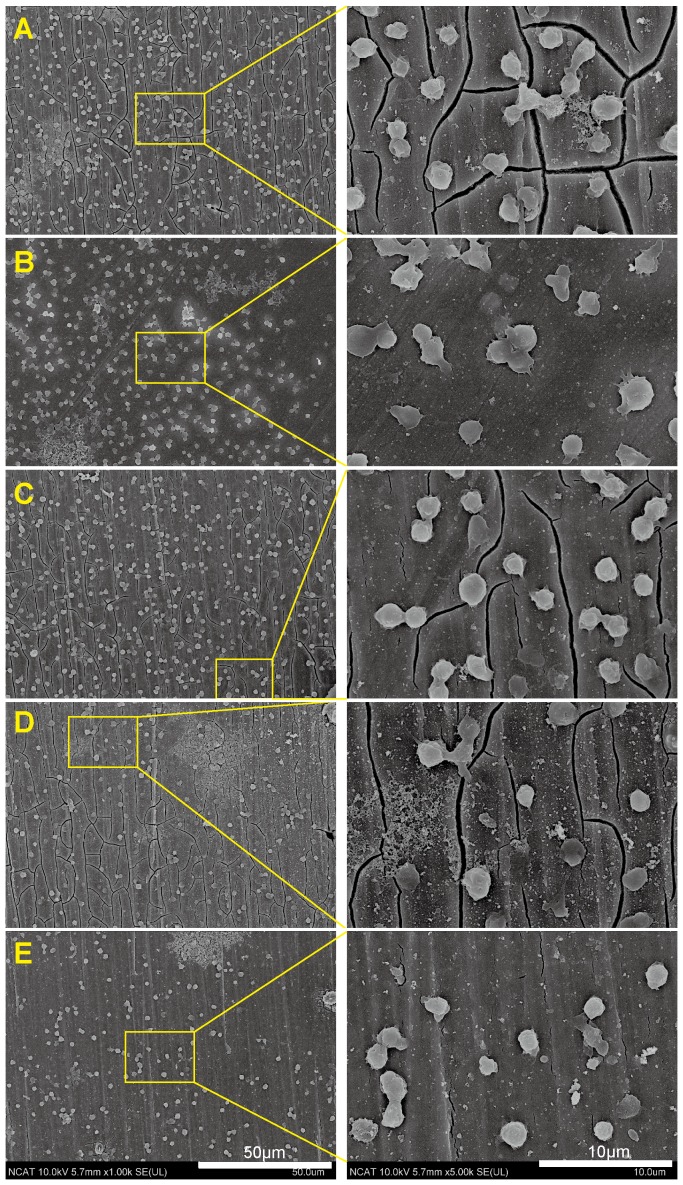
Representative SEM images of platelet adhesion and activation on Mg materials. (**A**) pure Mg, and (**B–E**) R1–R4.

**Figure 7:**
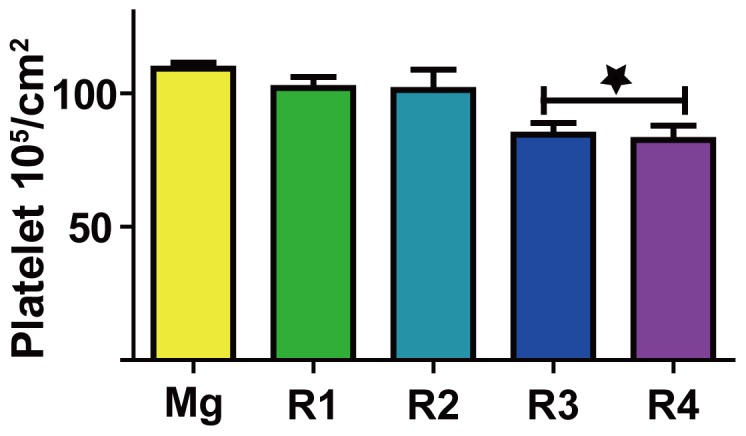
The number of adherent platelets on different materials calculated from SEM images. Star indicates that the platelet number is significantly different from that of pure Mg group (*P*<0.05).

The Fig. 6 showed the platelet adhesion and activation after incubation on the material surfaces for 1 hour. Activated platelets with spreading dendriticals connecting with their proximal platelets were observed on the pure Mg surface. In addition, a few platelets with long dentritical structure were seen under higher magnification. R1 and R2 had similar density of adherent platelets but with less spreading dendriticals compared to pure Mg. On the other hand, the number of adherent platelets on R3 and R4 was significant less than that on pure Mg (Fig. 7).

### Cell viability

**Figure 8:**
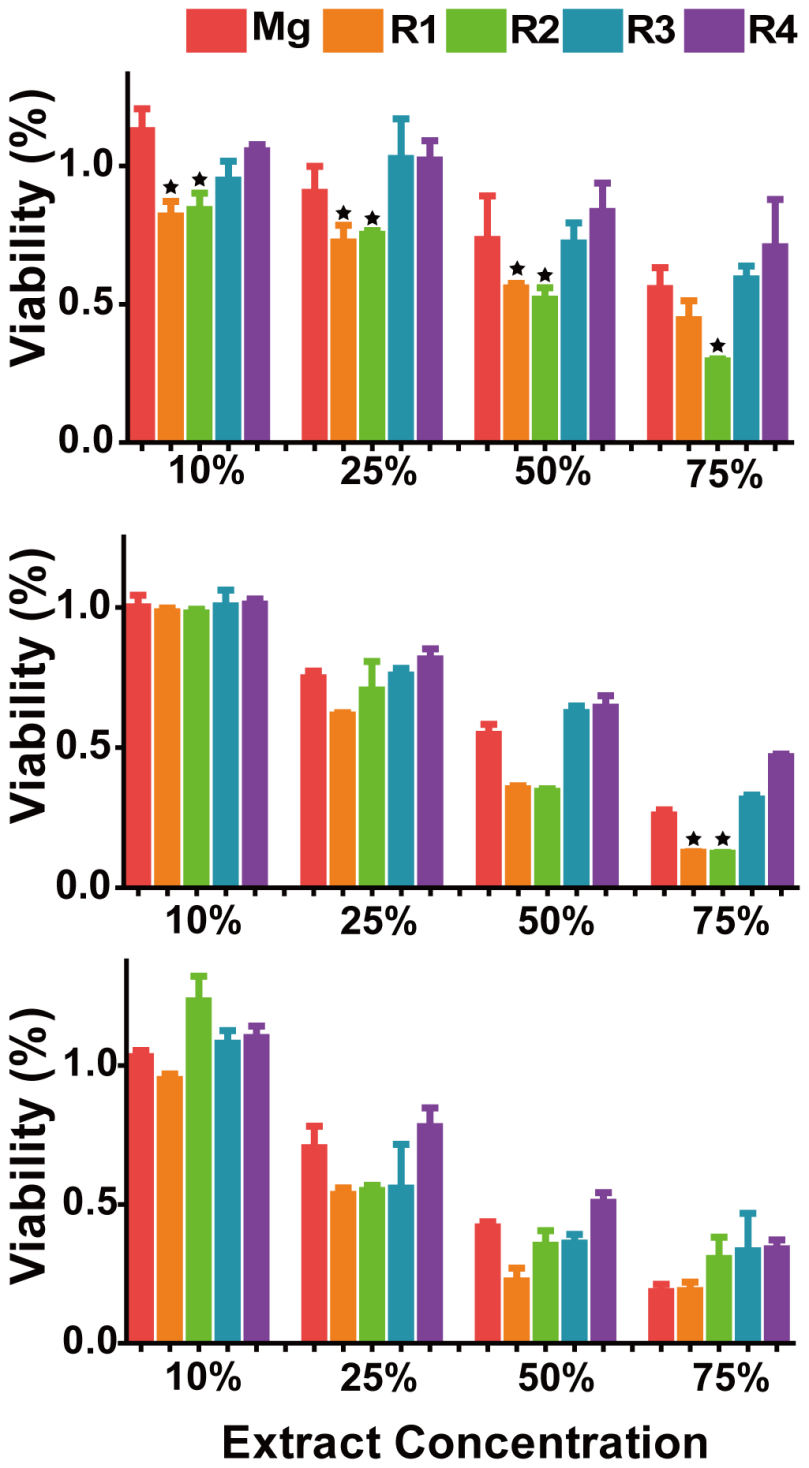
HAECs viability by MTT after treated with different Mg material extracts. (**A**) 2 days (**B**) 4 days, and (**C**) 7 days. Stars indicate that the cell viability is significantly different from that of pure Mg group (*P*<0.05).

**Figure 9:**
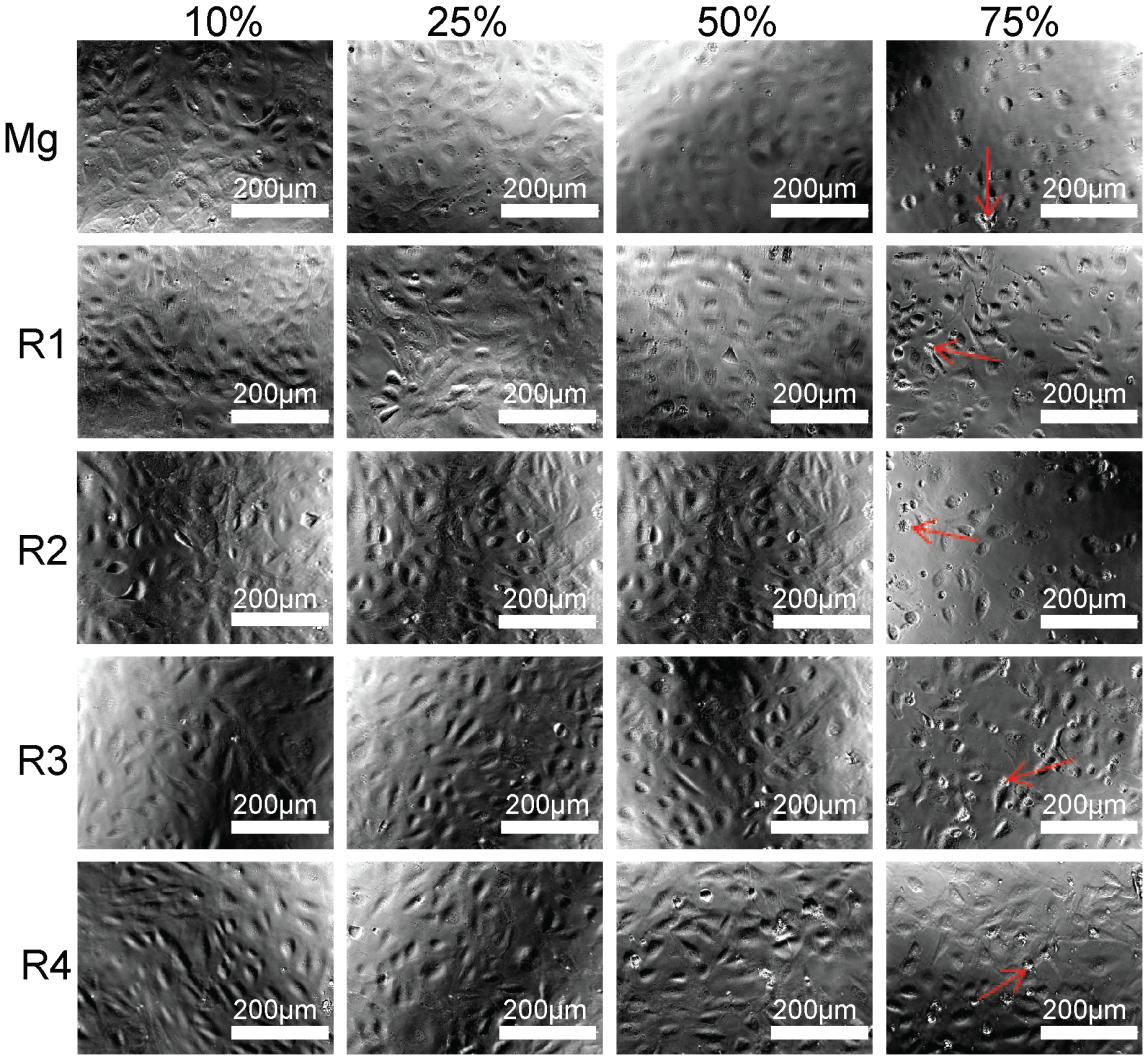
Representative optical images of HAECs morphology after treated with Mg material extracts for 7 days. Red arrow indicates the cell debris and dead cells.

Cell viability was usually tested by MTT in both a time course and a concentration gradient [35], [36]. Alloy extract solutions were prepared by incubating the materials with serum free ECM for 72 hours at 37°C with the material surface to volume ratio of 1.25 cm^2^/ml. Cell toxicity on days 2, 4 and 7 with different concentrations of alloy extract solutions was shown in Fig. 8. The overall cell viability for all groups decreased as the concentrations of the extract solutions increased. Extract solution of R3 and R4 didn't affect cell viability at any concentrations compared with pure Mg. On the 2^nd^ day, reduced cell viability was observed in R1 and R2. On day 4, there was no significant difference among all the materials and pure Mg control except for R1 and R2 at 75% concentration. On day 7, cell viability of all alloys was not significantly different from pure Mg control. Representative optical images of HAECs on day 7 were shown in Fig. 9. Cells all looked healthy, and there were no obvious morphological changes for all groups at 10% and 25% extract solutions. There were some dead cells and debris floated around when the extract concentration reached to 50%. In 75% group, the cell density decreased significantly, especially for the pure Mg.

### In vitro endothelialization

**Figure 10:**
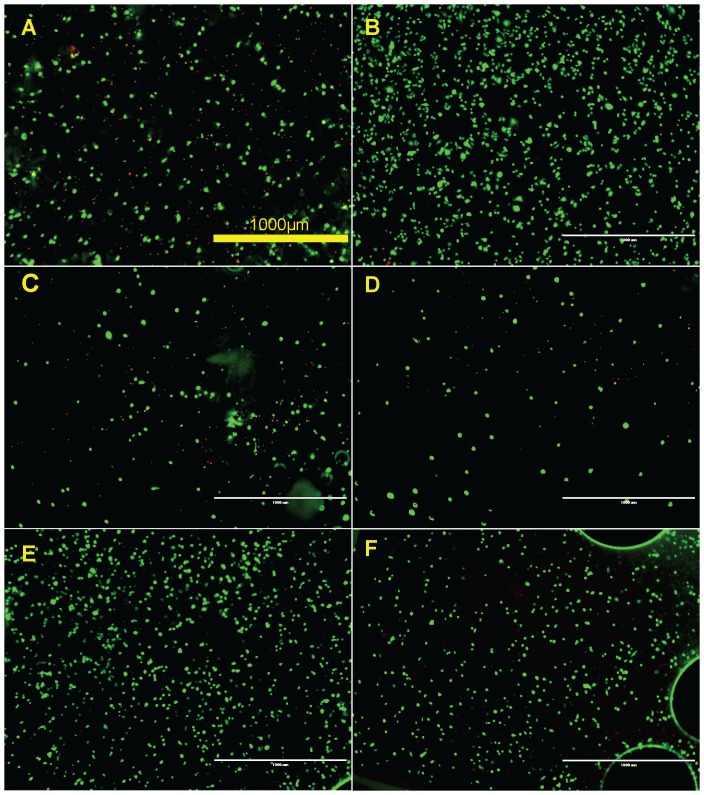
Representative fluorescent images of HAECs on Mg materials after 3 (**A**) Pure Mg, (**B**) Tissue culture plate, and (**C–F**) R1–R4. Live cells are in green and dead cells are in red.

Endothelial cell coverage is an important factor to measure the biocompatibility of a stent material. Endothelial cell attachment was studied by directly seeding endothelial cells on the material surface with tissue culture plate as positive control. The attached HAECs were detected by fluorescent staining with green for live cells and red for the dead. The density of attached cells after 3 hours on R1 and R2 were lower than that on pure Mg, tissue culture plate, R3 and R4 (Fig. 10). Most of the cells attached on the sample surface in all tested materials were still in round shape. Some dead cells could be observed on pure Mg, R1, and R2. In comparison, a few completely spread cells in healthy shape were seen on tissue culture plate, R3 and R4.

**Figure 11:**
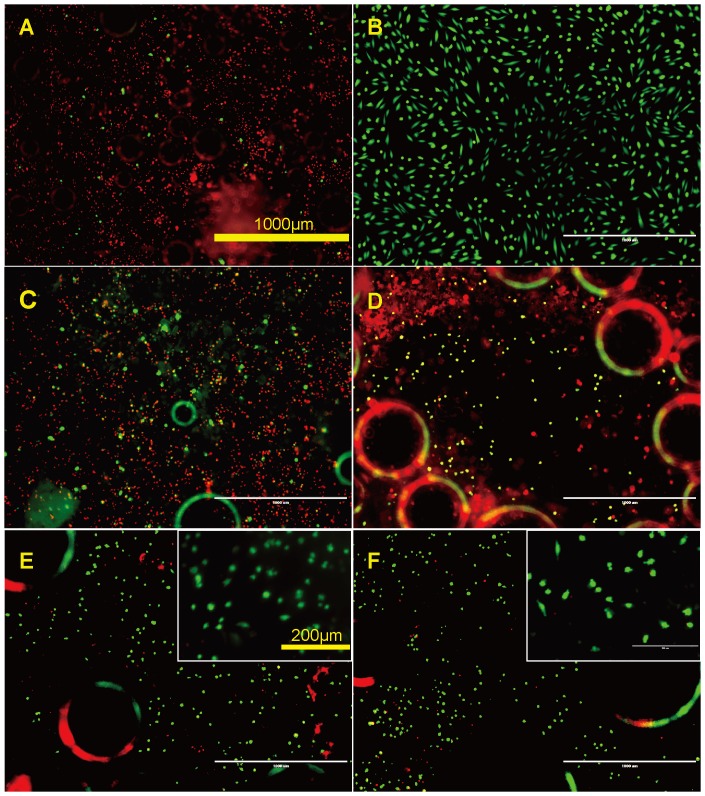
Representative fluorescent images of HAECs on Mg materials after 24 (**A**) Pure Mg, (**B**) Tissue culture plate, and (**C–F**) R1–R4. Live cells are in green and dead cells are in red.

**Figure 12:**
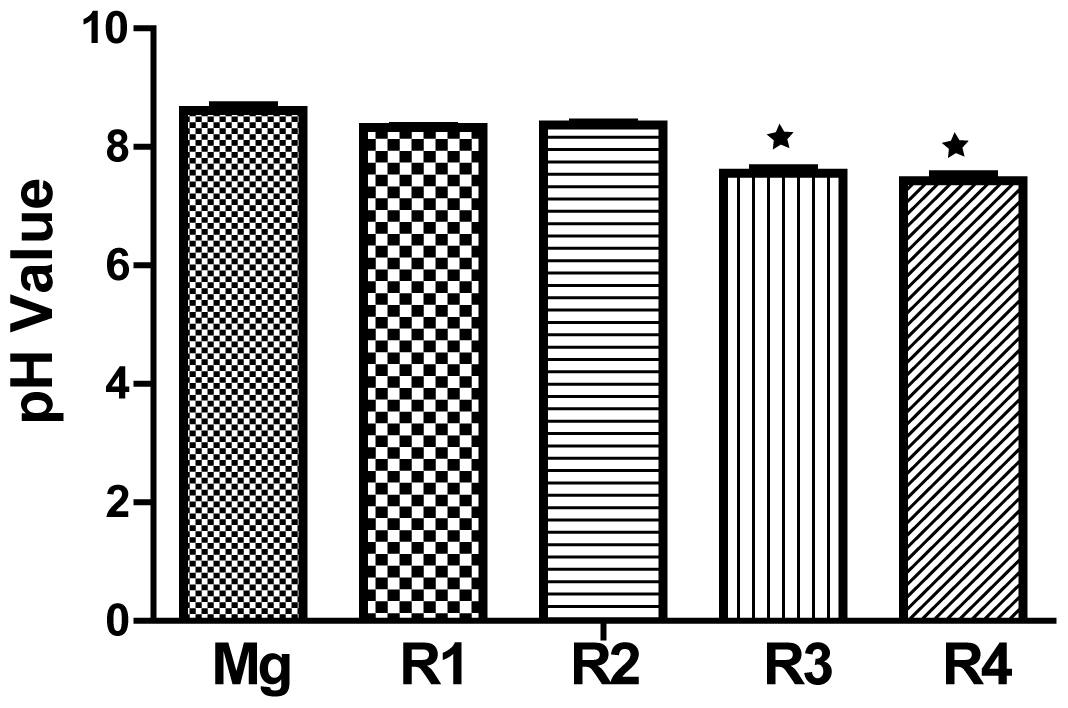
The pH value of serum free ECM after incubation with cells and Mg materials for 24 Stars indicate that the pH value is significantly different from that of pure Mg group (*P*<0.05).

After 24 hours, most cells on pure Mg were dead, and many air bubbles with diameter of ∼50 µm evolved on the surface of all the materials (Fig.11). More cells survived on R1 and R2 compared to pure Mg but were in a stressed condition. On R3 and R4, majority of the attached cells were still viable, and they looked healthy and well attached, spreading in a spindle shape. However, cell density on R3 and R4 significantly decreased compared to that on the tissue culture plate control. The pH value of the culture media after 24 hours was shown in Fig. 12.

## Discussion

### Alloy microstructure and mechanical properties

Due to the necessity to provide continuous mechanical support for the narrowed blood vessel during the entire course of vessel healing, sufficient mechanical strength is required for a good stent material. Mg alloys have special advantages over other biodegradable polymers for stent materials because of its higher tensile strength and modulus [37]. It is known that RE can refine grain structure in Mg alloys and improve the mechanical properties [38], [39]. Addition of RE elements had significant effects on improving the Modulus, YS, and UTS of Mg alloys (Fig. 2). Compared with the Mg-Ca alloy system developed by Li et al., the mechanical strengths and elongation of our RE alloys are at least twice as high as that of their as-cast Mg-Ca alloy [40]. The mechanical strengths of the four RE alloys are comparable to that of commercially available WE43 alloy [41]. The improved mechanical strength could be attributed to grain refining effect of REs (Fig. 1). The grain size in all our RE alloys were refined by RE at different degrees. Those intergranular secondary phases in R1, R2, and R4 may contribute to the corrosion resistance of alloys [38]. In R3, the reduced number of second-phase and increased grain size probably were caused by presence of Ca and some RE elements [42]. Substantial amount of second phases in R4 could also be caused by the presence of certain RE elements. In addition, for most balloon expansion stent, high elongation is necessary to allow enough stent dilation to a proper shape without breaking. R3 had the lowest elongation rate (∼11.25%) in all alloys, which could be explained by large and non-homogeneous grain size [43]. The elongation of R1 and R2 was high enough for the stent expansion process where 20% to 30% elongation is needed [44].

### Elecrochemical corrosion properties

Mg alloys as stent materials are expected to provide sufficient mechanical support for narrowed blood vessels over a few months. The gradual degradation of Mg stent by corrosion and subsequent body absorption could eliminate the long-term restenosis and avoid a second surgical operation for stent removal. Therefore, the corrosion rate is also an important factor to evaluate a stent material besides its mechanical properties. Unfortunately, clinical trials showed that current Mg-based stents were degraded too fast to enable the blood vessel to reprogram and heal completely [45]–[47]. Fast degradation will not only lead to reduced mechanical strength but also cause higher local pH, high alloying metal ions concentrations and too much degradation debris. In this study, the microgalvanic boundary corrosion in R1 and R2 may be attributed to the presence of RE intermetallic structure [48]. The RE forming complex intermetallic phases may generate galvanic circuit between the grain boundary and surrounding phases when the alloys were immersed into HBS [48]. This galvanic circuit could lead to the increased corrosion of Mg near the grain boundary. However, in another research it was suggested that some secondary phases can act as corrosion barrier and ameliorate the progress of corrosion [49], which is in agreement with the R4 case where a lot of secondary phases were present.

### Hemocompatibility

Platelet morphological and biochemical changes are good indicators for hemocompatibility [50], [51]. At the beginning of stent development, 20% of self-expandable stents would suffer from subacute stent thrombosis [52]. Platelet activation and adhesion are the important initial steps of restenosis [53], [54]. Individual platelets can be categorized as round, dendritic, spread-dendritic and fully spread corresponding to different stages of activation. Static platelet adhesion test is the most convenient and accurate way to get information about whether the stent material will cause severe platelet adhesion in vivo. Because extracellular Mg ions can reduce the intracellular calcium ions, Mg alloys could inhibit platelet adhesion and aggregation [55]. We showed that the density of attached platelets on pure magnesium was the highest, and the morphology of the adherent platelets on all materials was almost the same, demonstrating that addition of RE elements into Mg alloys didn't trigger the platelet activation. It is still unclear how platelet activation was initiated. We speculate that the unspecific absorption of von Willebrand factor (vWF) and other plasma protein caused the initiation of platelet adhesion. Circulating platelet can bind to vWF through their GPIb-V-IX receptor and lead to the activation cascade [56].

Hemolysis rate was not affect by adding RE elements, indicating the release rate of those RE elements was very slow and didn't reach the threshold causing severe red blood cell lysis. In fact, the hemolysis rate of R1 and R2 was even lower than that of pure Mg. The hemolysis effect was most likely caused by the degradation of Mg, and subsequent increased osmosis pressure and elevated pH level.

### Endothelial cytocompatibility

Re-endothelialization on stent material surface was suggested to be the key to reduce platelet adhesion, stent thrombosis and other adverse outcomes [57], [58]. It was shown that increased endothelial coverage could significantly improve the long-term patency and reduce the interaction of blood components with artificial implants [59]–[61]. Some studies used L-929 and MG63 cells to test the biocompatibility of Mg alloys [5], [30]. Little was known about how endothelial cells would interact with Mg alloys. In this study, Indirect MTT showed that there was a decreased cell viability tendency in all group as the increase of concentrations of extract solutions. This was most likely due to the higher pH level (Fig. 12) and degradation products, leading to mitochondrial oxidative stress. It was also interesting that no further decrease in viability were observed for R1 and R2 on days 4 and 7. In R3 and R4, cell viabilities through all the seven days were not significantly different from pure Mg, which indicated the release of REs from these two alloys didn't affect the HAECs viability. In addition, Fig. 9 showed even on the 7^th^ day, more than half of the cells were still in healthy morphology, which seems to contradict with the 7^th^ day MTT test. One possible explanation is that extract solution didn't cause lethal damage to cell membranes or genetic substances but only lead to decreased enzymatic activities or altered gene regulation.

In direct cell attachment test, R1 and R2 had the lowest density of attached cells after 3 h indicating the surface of those materials were least favorable for cell adhesion and attachment. This is most likely caused by the combinative effect of the presence of Dy and relative higher degradation rate when compared to R3 and R4. As the degradation of R1 and R2 progressed in ECM, Dy ions may inhibit the attachment of endothelial cells at the initial stage. Also, some swollen cells with green color were present on R1 and R2 indicating cells were dying though the cell membranes were still intact, therefore, retaining the green fluorescence. R3 had the comparable cell attachment and viability with the tissue culture plate control while R4 and pure Mg had moderate cell attachment. After 24 hours of incubation, most cells were dead on pure Mg and many air bubbles emerged. A few live cells could still be observed on R1 and R2, but obviously in stressed conditions. In contrast, fully spread cells were the major population on R3 and R4 surfaces but the density were much smaller than that on tissue culture plate. Among all the serum free ECMs samples incubated with Mg materials and cells for 24 h, the one with pure Mg had the highest pH change which would be the strongest stress to cells (Fig. 12). The death of cells could be mainly caused by increased pH value as the degradation of Mg alloys progressed. Results demonstrated that all Mg-RE alloys exhibited better endothelialization than pure Mg control in the static culture environment. It would be a totally different story in dynamic system or in vivo, and we expect that endothelial cells would have much better attachment, survival and growth in vivo as the dynamic circulation system will remove the degradation products and prevent the hike of local pH.

## Conclusions

Four novel Mg-RE alloys were successfully fabricated and tested for the feasibility as vascular stent materials with pure Mg as control. The microstructure, mechanical properties, corrosion resistance, hemocompatibility, and endothelial cytocompatbility were systematically evaluated. The microstructures of all four materials were significantly refined after the addition of RE elements. All the mechanical properties were remarkably improved. The corrosion rate was reduced at least 3 to 10 times than that of pure Mg control. Surprisingly, the addition of RE elements in Mg didn't bring any significant deleterious effects on hemolysis and the platelet adhesion, instead reduced platelet adhesion and activation were observed on all RE alloys. Indirect MTT test revealed that there was no significant difference between cell viability in RE alloys and pure Mg control in the static culture system. Direct endothelialization test showed that RE alloying could significantly improve cell attachment and spreading on the alloy surfaces. Taken together, these Mg-RE alloys are demonstrated with promising features as stent materials, and future in vivo studies are needed to fully assess their potential for cardiovascular stent applications.
